# Role of Intensive Training in Strengthening the Skills of HIV Counselors for Imparting Quality ICTC Services

**DOI:** 10.4103/0970-0218.55295

**Published:** 2009-07

**Authors:** D Dhadwal, AK Bhardwaj, AK Gupta, S Sharma, A Parashar, Anita Thakur, A Mahajan, V Chander, A Sood

**Affiliations:** Department of Community Medicine, IG Medical College Shimla, India

## Introduction

Counseling is a confidential dialogue between a client and counselor aimed at enabling the client to cope with stress and make personal decisions related to HIV/AIDS. When Voluntary Counseling and testing centers (VCTC) centers were started initially, their focus was on prevention of HIV infection, HIV testing, and dealing with social and emotional impact of a HIV positive test. The scope of services being provided has over the years expanded rapidly, with addition of prevention of parent-to-child transmission (PPTCT), access to anti retroviral therapy (ART), and better linkages with directly observed treatment short course (DOTS) centers.(1–4)

In the third phase of National AIDS Control Program (NACP-111), these VCTC services are being further expanded to Community Health Center (CHC) and Primary Health Center (PHC) levels and clients will be provided comprehensive, quality services under one roof of integrated counseling and testing centers (ICTC). This planned expansion puts pressure on NACP-111 to recruit and train a large number of qualified and skilled counselors who will be the most important functionaries of these VCTC's, PPTCT's, and ICTC's throughout the country.(5) The HIV counselor needs to equip the client to prevent HIV infection, to make an informed choice about HIV testing, to cope with an HIV test result and to understand the implications of lifelong treatment.(6)

National AIDS Control Organization (NACO) has developed an intensive 12 days training schedule for developing and fine tuning the counseling skills of these counselors so as to produce counselors who are sensitive to their clients problems, are well informed and can provide high quality VCT, PPTCT, and ART counseling. For this purpose, NACO has identified 18 institutes across the country who will impart trainings to HIV counselors. Our institute was selected to train counselors from Himachal and Haryana state. We tried to study the impact of 12-day intensive training program in enhancing the skills of these counselors.

## Materials and Methods

Two batches having 22 and 28 counselors respectively from Haryana and one batch having 32 counselors from Himachal Pradesh were trained by our institution from September to December 2007. All counselors were asked to fill a pre-training structured questionnaire to assess their knowledge before the start of the training program.

The training program consisted of seven modules which were further divided into sub modules with clearly stated objectives and session plans. Each sub module was covered according to session plan in detail using power point presentations and skill enhancing activities.

Since the counselors already had basic knowledge and skills of HIV counseling, more stress was given on participatory training to further improve and fine tune their counseling skills. The training strategy was interactive and involved trainees in practicing communication skills as well as developed their attitudes and skills for coping with fear, anger, and embarrassment. Each session of training involved strategies such as brainstorming, role plays, case studies, group discussions, and educational games.

A wide variety of topics were covered during the 12 days course of the training. Apart from re-emphasizing on basic topics such as VCTC issues, PPTCT, ART, and targeted intervention groups as Commercial sex workers (CSW), Men having sex with men (MSM), injectable drug users (IDU), additional topics were introduced to enrich the contents of training. These included crisis intervention and problem solving, group and family counseling, legal and ethical issues in HIV counseling. Various mental health issues faced by the clients which included suicide prevention and management of psychological distress were discussed in detail. A large number of expert faculties from other departments of Medical College as well as from State Health and family welfare training institute (SHFWTI) and state AIDS control society were empanelled for imparting the training.

Two field visits were also arranged to ICTC and ART centers located in the institution. The in charges of these centers and the counselors posted there provided hands on training to the participants. The counselors got an overview of the functioning of ICTC and ART centers attached to a tertiary care hospital.

At the end of the training course, the participants filled a post test questionnaire as well as a Performa for giving their feedback about the training program.

## Results and Discussion

Two batches consisting of 22 and 28 counselors each from Haryana and one batch from Himachal Pradesh with 32 participants were given 12 days induction training in our institute from the month of September to December 2007. First batch from Haryana had a total of 28 participants with equal proportion of both genders whereas in 2^nd^ batch of 22 counselors of Haryana, there were 12 males and 10 females. In the batch of counselors of Himachal Pradesh, total participants were 32, out of which 11 were males and 21were females. The participants were mostly young with age ranging from 21 to 35 years. Since all the participants in all three batches were fresh recruits with work experience ranging from one month to one year, more stress was laid on participatory learning techniques to further improve upon and fine tune their counseling skills. In each training, 10-15 role plays, 8-12 case studies, 5 brainstorming sessions were done. In addition, 5 educational games, two audio-visual demonstrations and two field visits to ICTC and ART centers were conducted. There were 18 mini lectures in the form of power point presentations delivered by different experts.

In the initial one or two days, the active participation was limited to a few experienced HIV counselors but gradually over next few days, all the participants involved themselves in various group discussions and role plays. Gradually with constant encouragement by the trainers, all the participants started taking keen interest and actively participated.

There was remarkable improvement in their knowledge as was evident from the significant difference between the pre test and post test scores. The average gain index for the three batches ranged between 33 and 37% [Table 1]. The difference of mean pre and post test scores for all the three batches were found to be statistically significant [Table 2]. The improvement in the quality of skills enhanced by the training was assessed by close observation of various participatory activities such as role plays etc. by the training faculty, having one to one interaction with the participants. Participants were seen raising queries and competing among themselves in the case studies and other educational games.

**Table 1:** Average pre and post test scores and Gain Index of HIV counselors

State	Number of counselors	Average pre test score (%)	Average post test score (%)	Gain index (%)
Haryana (Batch 1)	28	24	57	33
Haryana (Batch 2)	22	36	73	37
Himachal Pradesh	32	29	66	37

**Table 2:** Pre and post test scores

Batch	Pre test score Mean ± (s.d.)	Post test score Mean ± (s.d.)	*P* value
Haryana	25.09 (4.02)	50.64 (6.99)	< .001
Himachal Pradesh	19.91 (7.82)	45.22 (8.91)	< .001
Haryana	16.43 (3.26)	39.36 (8.41)	< .001

*P* value < .05 significant

On analysis of their feedback Performa's and by having discussions with the participants to know their views about the individual sessions as well as the program as a whole, it was evident that 90% of the trainees appreciated the training program and 85% said that they would recommend this program to other colleagues. Most of the HIV counselors felt that this type of training programs should become a regular feature so that they not only can update their knowledge but also fine tune and enhance their counseling skills.

Another important observation was that this type of training program gave them a platform to share their experiences with their colleagues and also to put forward their apprehensions and difficulties met by them during the counseling sessions, which were then allayed by the training faculty and the accompanying officials from state AIDS societies. The participants were specially encouraged by the introduction of additional topics such as crisis management and problem solving, mental health and management of psychological distress and suggested that such topics should be part of any future training curriculum. Almost all the participants, however, noted that this 12-day program was a little too exhaustive and requested if it could be squeezed to duration of one week. The probable reason for this response could be attributed to the element of home sickness for being out continuously for a fortnight. However, the organizers made all sincere efforts to make the training schedule as relaxing and enjoyable as possible by introducing a lot of educational games, activities and arranging for excursions during spare time without diluting the quality of the training.

## Conclusions

The conviction held by NACO is that the clients accessing a VCT, PPTCT or ART center need to understand the context of HIV/AIDS from prevention to treatment and care. Thus, the HIV counselors are challenged not only to keep abreast with new trends in HIV/AIDS prevention but to continually fine-tune their skills to address various needs of their clients in comprehensive and sustainable manner. This type of exhaustive training program not only standardizes HIV counselors training across the country but also allow the states to build the capacity of a very important human resource in the struggle against HIV/AIDS. These trainings can be followed up by having refresher trainings in the future so that the training institutions can help in providing ongoing support for counselors including mentoring, supervision, and monitoring.
